# Effect of Ferrule Location on Fracture Resistance of Maxillary Premolars: An In Vitro Study

**DOI:** 10.1155/2023/9513804

**Published:** 2023-08-09

**Authors:** Sara Koosha, Mahdieh S. Jebelizadeh, Azam S. Mostafavi

**Affiliations:** ^1^Department of Prosthodontics, School of Dentistry, Azad Islamic University, Tehran, Iran; ^2^Private Practice, Tehran, Iran; ^3^Department of Prosthodontics, School of Dentistry, Tehran University of Medical Sciences, Tehran, Iran

## Abstract

**Objectives:**

The present study aimed to assess the effect of ferrule location on fracture resistance of maxillary premolars.

**Materials and Methods:**

A total of 72 extracted human maxillary premolars were selected and randomly assigned to six groups (*n* = 12 in each) considering ferrule location: circumferential ferrule (CF), without ferrule (WF), buccal ferrule (BF), lingual ferrule (LF), mesial ferrule (MF), and buccal–lingual ferrule (BLF). Cast posts were cemented into the prepared post spaces. Following conventional impression, Ni–Cr crowns were cemented to the specimens. After thermocycling (5,000 cycles, 5–55°C), the specimens were loaded at 45° in a universal testing machine until fracture. Data were analyzed with one-way ANOVA, Kolmogorov–Smirnov, and Tamhane tests.

**Results:**

The maximum and minimum mean fracture resistance were related to the CF (1,143.84 N) and WF (514.89 N) groups, respectively, (*P* = 0.039). Fracture resistance in the BF (933.67 N) and BLF (874.01 N) groups was significantly higher than in the MF group (617.54 N) (*P* = 0.001). There was no significant difference between the MF, LF (722.89 N), and WF groups in terms of fracture resistance (*P* > 0.05).

**Conclusion:**

Teeth with CF showed maximum fracture resistance. The location of the ferrule effects on the fracture resistance of maxillary premolars and also the mode of failure.

## 1. Introduction

Coronal reconstruction of severely damaged teeth after root canal treatment is still challenging for many dental clinicians. Endodontically treated teeth (ETT) are often more susceptible to fracture due to great loss of coronal structure following extensive caries, previous restorations, and endodontic treatment [1, 2]. In case of more than 50% destruction of coronal structure, post and core restoration is recommended [3]. Evidence shows that the remaining coronal tooth structure is a more important influencing factor than the type of post-core system to resist fracture and durability of the restoration [4–7]. Ferrari et al. [8] declared the significant role of preserved coronal walls in reduced failure risk of endodontically treated (ET) premolars regardless of restorative plan. According to Pantaleón et al. [9] study, even increasing the height of remaining axial walls cannot compensate the absence of missing walls; which was confirmed by Zahran et al. [10] and Sherfudhin et al. [11]. Finite element studies have shown that the stress concentration is in the cervical region of ETT restored with post-crown and the ferrule effect has a positive role in reducing the stress level. Also, it has been reported that presence of ferrule results in favorable stress distribution in root [11–16]. The minimum required height for the remaining tooth structure should be 2 mm from the finish line to the tooth/core interface; it should be circumferential and include all four axial walls to provide a ferrule effect [17]. However, in the clinical setting, the clinicians may encounter cases in which the height and location of the remaining tooth structure are not uniform in all walls. Cervical abrasion, erosion, and abfraction are among the factors causing buccal wall degradation. Deep or secondary caries can lead to the loss of tooth structure and elimination of the ferrule effect in proximal areas. Also, subgingival preparation of the finish line (to provide greater esthetics) leads to a compromised ferrule, especially on the labial surface. Although crown lengthening is a viable solution in these situations, but it can adversely affect the esthetic, and also leads to complications, such as compromised crown/root ratio and traumatization of the adjacent teeth. Furthermore, forced eruption may not be feasible since it prolongs the treatment course and increases the costs [18]. Thus, it is important to better understand the role of the remaining tooth structure (considered as ferrule) and its location [10, 19] in the durability and longevity of restorations. Evidence in this context, can help dental clinicians in making a correct decision about the necessity of other treatments, such as orthodontic treatment or surgical crown lengthening to increase the success of treatment. Samran et al. [20] evaluated mandibular premolars and demonstrated that the location of ferrule had no significant effect on the fracture resistance. However, Ibrahim et al. [21] reported that a higher number of residual walls increased the fracture resistance of maxillary premolars; in addition, the presence of the palatal wall significantly increased the fracture resistance. Many studies evaluated the effect of the ferrule on fracture resistance of anterior ETT [22–26]. However, there are controversial results about the role of the amount and location of the remaining ferrule in premolars; lingual ferrule (LF) was highlighted to be more effective in some studies [18, 27], while Dua et al. [28] reported that buccal ferrule (BF) showed better results than other groups. Also, some studies presented nonsignificant results among the included groups [11, 20] indicating the need for further investigations. Thus, the present study aimed to assess the effect of existing ferrule location on fracture resistance in maxillary premolars. The null hypothesis was that teeth with different ferrule locations would present similar fracture resistance.

## 2. Materials and Methods

**Figure 1:**
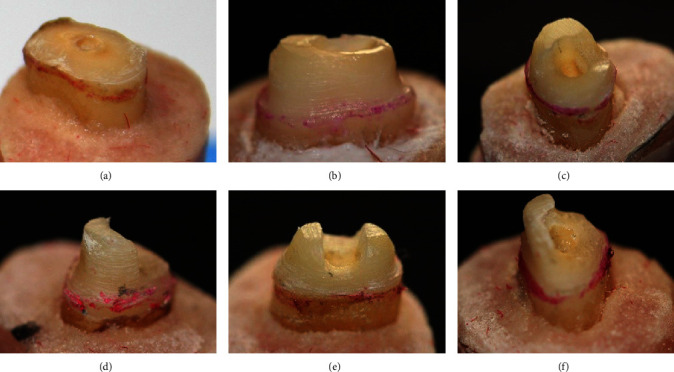
Preparation of different ferrule designs in test groups: (a) Without ferrule, (b) circumferential ferrule, (c) buccal ferrule, (d) lingual ferrule, (e) buccal–lingual ferrule, and (f) mesial ferrule.

Based on Samran et al. [20] study, using two-sample *t*-test in PASS 11 software, considering a significance level of *α* = 0.05 and *β* = 0.8, average standard deviation of 170 N for fracture strength in order to detect significant difference of 200 N, the sample size was determined *n* = 12 in each group. The present in vitro, experimental study evaluated 72 single-canal maxillary premolars extracted due to periodontal problems or as a part of orthodontic treatment plan. Teeth were immersed in a solution containing 0.9% physiologic saline with 1% thymol at room temperature immediately after extraction. All the study phases were conducted by the same operator according to the standard protocols. The teeth with carious lesions, microcracks, restorations, cervical abrasion, or anomalies were excluded. The root length was measured from the cementoenamel junction (CEJ) to the apex. Also, the buccolingual, mesiodistal, and occlusogingival dimensions of tooth crowns were measured by a digital caliper, and teeth with approximately equal dimensions (maximum of 10% difference) were enrolled. The crown portion of all specimens were cut 3 mm above the CEJ. To simulate the periodontal ligament, the roots surface was coated with an artificial periodontal membrane made of condensational silicone (Speedex Light Body C-Silicone, Coltene Co.) [20]. By using a surveyor, specimens were mounted in cylinders containing auto-polymerizing acrylic resin (Acropars, Marlic Co.) along their longitudinal axes. The working length was radiographically determined 1 mm above the apex. The root canals were then instrumented with #15–#45 hand files (Mani H-Files, 15–45 mm, Mani Co.). During instrumentation, the root canal was rinsed with 3% sodium hypochlorite; lateral condensation technique was performed using gutta-percha and AH-26 silver-free sealer (Dentsply Co.). The access cavity was temporarily sealed with temporary restorative material (Cavisol; Golchai Co.) and the specimens were immersed in distilled water at room temperature for 72 hr. Post spaces were prepared as two thirds of the root length using #2 and #3 Peeso reamers (Mani Co.). Afterward, teeth were randomly assigned into six groups (*n* = 12) as follows:

Circumferential ferrule (CF) group: the roots with ferrule in all axial walls with 2 mm height. Without ferrule (WF) group: the roots with no ferrule. BF group: the roots with 180° ferrule in the facial. LF group: the roots with 180° ferrule in the lingual surface. Mesial ferrule (MF) group: the roots with 180° ferrule in the mesial. Buccal–lingual ferrule (BLF) group: the roots with ferrule in the buccal and lingual surfaces (Figure 1).

The specimens were prepared so that all ferrules had 1 mm width and 2 mm height. A deep chamfer finish line with 0.8 mm width was also prepared 1 mm above the CEJ; to obtain the 6° standard taper in axial walls, the high-speed handpiece was attached to a custom made parallelometer. Post patterns were made using Duralay acrylic resin (Economy Co.) and cast with Ni–Cr alloy. Post-core restorations were cemented by glass ionomer cement (GIC, Luting and lining cement, GC Co., Tokyo, Japan) and impressions were made (Betasil, Vario-Muller Omicron GmbH) using a special tray with stops. Impressions were poured with Type IV dental stone (Aria Dent, Iran). Wax patterns were designed and milled (Ceramill Motion 2, Ceramill Amann Girbach) and after investing (Z4Universal investment, N75, Belgium), were cast with nickel–chromium alloy (MeganiumCS, Megadental GmbH) due to its common use and popularity in PFM restorations. The crowns were then cemented on the abutments with GIC. After cementation, each crown was placed in a piston and subjected to 29 N load with a custom-made device for 7 min. All specimens were stored in distilled water at 37°C for 24 hr and then subjected to 5,000 thermal cycles (Dorsa Co.) at 5–55°C with 20 s of dwell time and 20 s for transfer time. Compressive load was then applied in the universal testing machine (STM20; Santam engineering Design Co.) at a cross-head speed of 1 mm/min at 45° relative to the longitudinal axis of the teeth. The load was applied to a groove created in the middle of the palatal slope of the buccal cusp until the fracture occurred. The fracturs were classified as restorable and nonrestorable according to Sulaiman et al. [27] study. The normality of data distribution was evaluated by the Kolmogorov–Smirnov test, and the results revealed a normal distribution of data in all groups (*P* = 0.2). Data were analyzed by one-way ANOVA followed by the Tamhane test. A *P*-value of <0.05 was considered statistically significant.

## 3. Results

**Figure 2:**
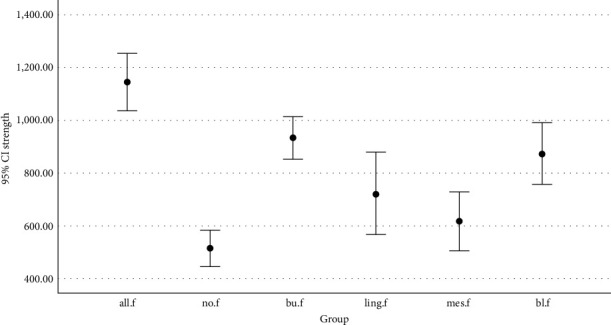
Bar chart of the mean and standard deviation of fracture resistance of the groups.

One-way ANOVA results showed that the CF and WF groups had the maximum (1143.84 N) and minimum (514.89 N) mean fracture resistances, respectively; there was a significant difference in fracture resistance among the groups (*P* < 0.05) (Table 1). Therefore, Tamhane test was used for pairwise comparisons, and the results showed a significant difference between the CF and other groups regarding the mean fracture resistance (*P* < 0.05). In general, the fracture resistance of the WF group was lower than those of the other groups; however, the difference was insignificant with those of the LF and MF groups. The BF group had the highest fracture resistance among the segmental ferrule groups; however, it only had a significant difference with the MF group (*P* = 0.001) (Table 1, Figure 2).

Assessment of the failure mode revealed that catastrophic or nonrestorable failure occurred in the root in all groups; although the frequency of failure location was different in the tested groups (Table 2).

## 4. Discussion

ETT are vulnerable to fracture due to losing a large portion of tooth structure. According to the evidence, the residual coronal tooth structure is a more important factor than the type of post-core system and also the length of the post to resist the applied loads [4, 26, 29]. Casting post cores were selected in this study due to their higher fracture resistance compared to other types [30]. Also, they can be formed according to root canal spaces which provide passive fitness and more even stress distribution [31]. Although irreparable fractures have been reported as a consequence in some occasions, but they have shown an over of 19.5 years survival rate [32, 33]. According to finite element studies, presence of ferrule yields favorable stress distribution in roots. In addition, higher ferrule would result in lower stress and consequently decrease in clinical failure [12, 13, 15, 34]. It is highly favorable to provide a complete CF with a minimum height of 2 mm which improves the prognosis of ETT [29]. However, such condition cannot be always obtained in the clinical setting, and the ferrule may be incomplete due to caries extension or fractures structure. The obtained results indicated the maximum mean fracture resistance in the CF group (1,143.83 N) and minimum fracture resistance in the WF group (514.89 N). This finding highlighted the significant role of ferrule in the reduction of stress distribution to the tooth structure and confirmed the previous findings in this context [2, 3, 7, 35]. Also, the current results revealed that the fracture resistance of the BLF and BF groups was significantly higher than that of the WF group while there were no significant differences among LF, MF, and WF groups in terms of fracture resistance. In other words, teeth with coronal structure in buccal surface or buccal and lingual walls increased the mean fracture resistance to 933 and 874 N, respectively, which were almost twice higher than the values in specimens without a ferrule. Therefore, the null hypothesis was rejected. Studies have shown higher fracture resistance in teeth with CF than those without it which means that the presence of circumferential residual tissue results in better stress distribution in tooth structure. Samran et al. [20] evaluated the role of the segmental ferrule in the buccal, lingual, and buccal–lingual surfaces in mandibular premolars, and showed that the location of ferrule had no significant effects on fracture resistance. Also, Figueiredo et al. [36] revealed even no significant difference between groups with segmental ferrule (BF, LF, and BLF) and CF group which was justified by the using fiber glass as post material and fatigue test. According to previous studies [20, 36], it appears that the location of ferrule plays no significant role in increasing the fracture resistance of ET mandibular premolars. The result of the current study was in agreement with the previous studies about the similar segmental ferrule groups, although in the present study MF revealed lower fracture resistance compared to BF and BLF which may emphasize on the more prominent role of buccal and lingual walls rather than proximal walls. At the same time, Sulaiman et al. [27] concluded that the LF plays a more important role in mandibular premolars than other areas. Different test conditions, such as the direction of force application and the type of post-core system, are probably effective in the results. In agreement with Sulaiman et al. [27] findings, other studies accentuated the positive role of LF on fracture resistance of ETT. Although in recent researches, anterior teeth were evaluated and loaded on the palatal side [6, 37, 38].

Regarding maxillary anterior teeth, it is recommended that the presence of a palatal ferrule is of great importance, because applying forces to the palatal side at an angle of 135° to the long axis of the tooth causes tension in the palatal tissue due to the crown's arc of displacement which supports the post-core assembly [39]. This issue can be generalized for posterior teeth as well. In the present study, since the application of force was on the palatal slope of the buccal cusp, 45° to the long axis, tension was created in the buccal tissue which maintains the crown and prevents its arc of displacement. Generally, since the direction of applying force in the posterior teeth, is mainly on the slope of the buccal and lingual cusps, presence of tooth tissue toward the applied force direction, prevents the arc of displacement in the restoration. In this regard, it seems that buccal and lingual walls are more important than proximal walls for posterior teeth. In this regard, Ausiello et al. [40] study showed that the maximum stress levels were detected in the palatal wall of canines. In addition, the findings of the current study align with those of Xing-Ming and Feng-Ming [41] who determined that the fracture resistance increased as the dentin wall of the ferrule approached the applied force. In line with the obtained results, Dua et al. [28] concluded that BF resulted in higher fracture resistance compared to LF and BLF; the specimens were loaded on the buccal cusp similar to the present study. Without enough dental tissue toward the direction of oblique forces, the arc of displacement in the restoration would results in bond failure between the post-core and dental tissue, and then the tooth structure may undergo the fracture. In the present study, the recorded fracture loads for LF and MF groups were less than the BF and BLF groups (Table 1).

Al-Wahadni and Gutteridge [42] evaluated the effect of the partial ferrule of maxillary anterior teeth on fracture resistance and reported that the teeth with a facial ferrule of 3 mm height had a higher fracture resistance than those without a ferrule. On the other hand, Naumann et al. [43] and Tan et al. [44] evaluated the effect of a 180° partial ferrule on fracture resistance in the buccal, lingual, and proximal surfaces of anterior teeth and pointed to the pivotal role of the proximal wall in fracture resistance of anterior ETT. They added that the teeth without proximal walls undergo fractures under smaller loads. However, the position of the tooth in the dental arch, type of tooth, and the direction of load application are among the factors influencing the results.

Ibrahim et al. [21] observed that as the number of residual walls increased in ET maxillary premolars, the fracture resistance of the teeth increased as well. Teeth with residual palatal walls showed significantly higher fracture resistance and the authors explained it based on the direction of the applied load which was against the palatal incline of the buccal cusp. Due to the fulcrum on the buccal side, the remaining palatal tissue would resist the crown's displacement; the difference could be related to the treatment plan in which the specimens were rehabilitated by composite core without post. Caplan et al. [4] showed that ETT with no or one proximal wall were weaker than those with two proximal walls. Thus, preservation of marginal ridge, if possible, is highly important. However, the location of the tooth in the arch, the type of post-core system and the amount of remaining dental tissue are factors that influence the results of studies. Since only MF was evaluated in this study, it would be valuable to investigate the results in presence of both proximal walls in future studies.

The maximum bite force in the first and second premolars is 178.5 ± 77.2 and 206.01 ± 86.52–N in females and 254.08 ± 72.20 and 291.36 ± 79.29–N in males, respectively [4]. However, it should be noted that the actual load required in vitro for failure may be higher than that of in vivo. In the current study, the load required for fracture was 1143.84 N in the CF group. This value ranged from 514.89 to 933.67 N in other groups which were higher that maximum bite force in the oral cavity. On the other hand, in vitro tests cannot simulate the clinical conditions perfectly because, in the oral cavity, teeth are subjected to shear, tensile, and compressive loads which have dynamic nature in a humid environment. Another limitation of the current study was the lack of aging process.

The failure mode was also evaluated in the present study. The results indicated root fracture was catastrophic and nonrestorable in all specimens. In general, cast posts lead to greater stress accumulation at the post-dentin interface. Since the modulus of elasticity of the cast posts is higher than that of dentin; fracture occurs in dentin. The presence of ferrule in the coronal region leads the stresses to be transferred to an area farther from the ferrule location. Consequently, fractures mainly occur in the middle third of the root in the CF group. In teeth with lower fracture resistance, the fracture line was closer to the cervical part of the root and CEJ [26]. Stress analysis of ETT with ferrule has revealed reduced stress levels at the cervical region in some studies [45, 46] which coroborates the obtained results of this study. Although others reported the shift of Von mises stress from the midroot and apex to the cervical region [14, 47]. In the current study, fracture occurred mainly in the middle third of the roots in the BF and CF groups. In the WF, LF, and MF groups, fracture more commonly occurred in the cervical third of the root. In this regard, Zhang et al. [48] reported that fracture location was transferred from dentin-core interface to dentin-post interface in specimens with higher damage load. Considering the limitations of invitro studies, future prospective clinical trials are required to validate the results of the present study.

## 5. Conclusion

Within the limitation of this in vitro study, the specimens in the CF group indicated the maximum fracture resistance. The location of ferrule was effective on fracture resistance of ET premolars and their mode of failure. Regarding less fracture resistance in MF group, teeth with only MF could be more prone to failure. The location of root fracture was in the cervical third of the root in teeth with lower fracture resistances and the middle third of the root in those with higher fracture resistances.

## Figures and Tables

**Table 1:** Mean fracture resistances and SD_s_ of all groups using one-way ANOVA (*N*).

Groups	Min	Max	Mean ± SD	*P*-value
CF	918.00	1,438.00	1,143.84 ± 170.97^a^	
WF	362.60	687.00	514.89 ± 108.84^b^	
BF	719.90	1,116.00	933.67 ± 126.04^c^	
LF	404.10	1,066.00	722.89 ± 245.30^bc^	<0.001
MF	394.30	943.40	617.54 ± 174.47^b^	
BLF	570.00	1,126.30	874.01 ± 183.56^c^	

C, circumferential ferrule; W, without ferrule; B, buccal ferrule; L, lingual ferrule; M, mesial ferrule; B, buccal–lingual ferrule; same superscript letters show mean values with no statically significant difference between groups (*P* > 0.05).

**Table 2:** Location of fracture and its frequency percentage in tested groups.

Group	Location of fracture	
	Root cervical third	Root middle third
CF	(4) 33.33%	(8) 66.66%
WF	(9) 75%	(3) 25%
BF	(5) 41.6%	(7) 58.4%
LF	(9) 75%	(3) 25%
MF	(9) 75%	(3) 25%
BLF	(7) 58.4%	(5) 41.6%

## Data Availability

Data supporting this research article are available from the corresponding author or first author on reasonable request.
